# Chromosomal imbalances in nasopharyngeal carcinoma: a meta-analysis of comparative genomic hybridization results

**DOI:** 10.1186/1479-5876-4-4

**Published:** 2006-01-19

**Authors:** Xin Li, Ena Wang, Ying-dong Zhao, Jia-Qiang Ren, Ping Jin, Kai-Tai Yao, Francesco M Marincola

**Affiliations:** 1 Immunogenetics Section, Department of Transfusion Medicine, Clinical Center, National Institutes of Health, Bethesda, MD, 20892, USA; 2 Department of pathology and Cancer research Institute, College of Basic Medicine, Southern Medical University, Guangzhou 510515, Guangdong Province, PR. China; 3 Biometric Research Branch, Division of Cancer Treatment and Diagnosis, National Cancer Institute, National Institutes of Health, Bethesda, MD, USA

## Abstract

Nasopharyngeal carcinoma (NPC) is a highly prevalent disease in Southeast Asia and its prevalence is clearly affected by genetic background. Various theories have been suggested for its high incidence in this geographical region but to these days no conclusive explanation has been identified. Chromosomal imbalances identifiable through comparative genomic hybridization may shed some light on common genetic alterations that may be of relevance to the onset and progression of NPC. Review of the literature, however, reveals contradictory results among reported findings possibly related to factors associated with patient selection, stage of disease, differences in methodological details etc. To increase the power of the analysis and attempt to identify commonalities among the reported findings, we performed a meta-analysis of results described in NPC tissues based on chromosomal comparative genomic hybridization (CGH). This meta-analysis revealed consistent patters in chromosomal abnormalities that appeared to cluster in specific "hot spots" along the genome following a stage-dependent progression.

## Background

Nasopharyngeal carcinoma (NPC) is a Epstein-Barr virus-associated cancer [1] highly prevalent in Southeast Asia and especially southern China where it occurs at a prevalence about a 100-fold higher compared with other populations not at risk [2,3]. Although environmental factors may contribute to this geographical association [4], the high prevalence of NPC in U.S. immigrants suggests a genetic influence [5]. Indeed, genetic markers associated with the disease have been proposed including an enhanced or reduced prevalence of some HLA haplotypes [6] or, more recently, a genomic region within chromosome 4 identified through genome-wide scanning of case control studies of familiar NPC from the Guangdong region [7].

Independently of the genetic root at the basis of its geographical prevalence, NPC is progressively characterized by widespread genomic imbalances possibly occurring before and during carcinogenesis [8]. Different strategies of comparative genomic hybridization (CGH) have been utilized to screen NPC for evidence of consistent chromosomal gains and losses along the progression of the disease [9-14]. However, most studies included a relatively small number of cases applied to different experimental models including the analysis of cell lines, xenografts or neoplastic tissues impairing the ability to interpreter the significance and evaluate the true frequency of chromosome imbalances in NPC. In particular, stratification of cases according to staging could not be performed at a power that could yield statistically meaningful information due to the limited sample population included in individual studies. Therefore, we entertained a meta-analysis of available information based on CGH of NPC samples derived directly from neoplastic material and excluding information derived from cell lines which might skew the true *in vivo *prevalence although others have shown a reasonable correlation between genetic alterations observed *in vivo *and those identifiable through the study of cell lines [15,16].

Six studies were identified that utilized directly tissue samples and contained sufficient information to allow a direct cross comparison of the results among them [8-12,17]. In this fashion, 188 NPC cases from Southern Asia were evaluated for which the CGH-derived information could be related to clinical staging.

## Results

### Chromosomal imbalances in patients with NPC

**Figure 1:**
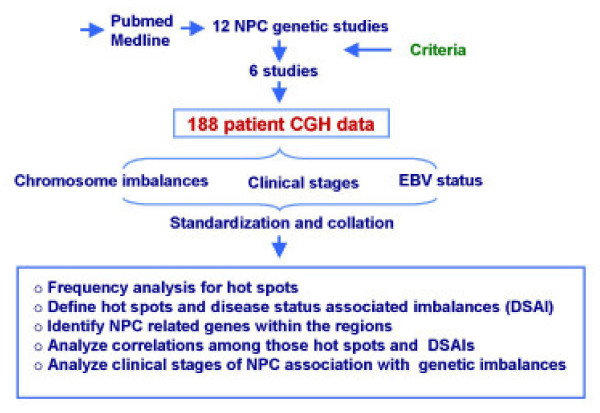
Flow chart summarizing the strategy adopted for the meta-analysis of chromosomal imbalances associated with NPC. The analysis was based on 6 studies whose information was comparable and reported on tissue samples eliminating studies reporting on cell lines or xenograft information [9-12, 17, 51]. See Materials and Methods for details.

**Figure 2:**
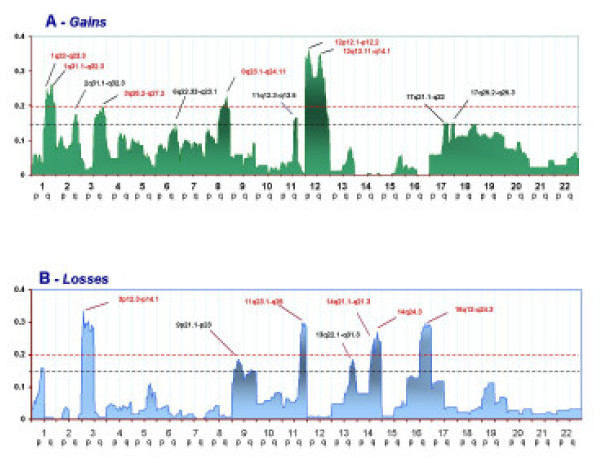
Frequency of chromosomal gains (panel A) or losses (panel B) in 188 samples derived from patients with NPC. The Y axis reports the frequency of chromosomal imbalances for individuals chromosome regions clustered into sections as described in the Materials and Methods. The X axis represents and ordered distribution of the chromosome analyzed. A cut-off of 0.15 corresponds to a frequency of imbalances higher than 15% arbitrarily selected to define "hot spots". A cut-off of .20 defines the predominant hot-spots used for clustering analysis.

The flow chart of the strategy applied for the meta-analysis is summarized in Figure 1 (see also materials and methods). The complete meta-analysis of all available CGH data revealed that chromosome gains were prevalent in 1q, 2q, 3q, 6q, 8q, 11q, 12p, 12q, 17p, and 17q (Figure 2). Hot spots could be identified in several regions: the ones with highest prevalence were in 1q, 3q, 8q, 12p, and 12q displaying more than 20% frequency. Hot spots with slightly lower prevalence (15 to 20%) could be identified in, 2q, 6q, 11q, 17p, and 17q. Chromosome losses occurred mainly on 3p, 8p, 11q, 13q, 14q, and16q. Among them, losses in 3p, 11q, 14q, and 16q occurred with more than 20% frequency while in 8p and 13q occurred with 15%-20% frequency. In total 12 hot spots were identified by this meta-analysis. Known oncogenes, tumor suppressor genes or other NPC related genes located in these hot-spots are presented in Table 1.

**Table 1:** Genes located in "hot spots" identified by the present meta-analysis

**Ch**	**Arm**	**Host spot, DSAI**	**Freq (%)**	**Candidate NPC-related Genes**	**references**
1	+1q	+ 1q22-q23.3	25.00	LAMC2	[13]
		+ 1q31.1-q32.3	26.06		
2	+2q	+ 2q31.1-q32.3	17.55		
3	-3p	- 3p12.3-p14.1	33.51	FHIT	[31]
		- 3p21.2-p21.33	30.32	RASSF1A,, Blu	[32,33]
		- 3p24.2-p26.3	29.26	RARβ2, RAF1	[13,34]
	+3q	+ 3q26.2-q27.2	19.68	deltaN-p63	[35]
6	+6p	+6p21.2-p23	23.33 E	HSP70-2	[36]
8	+8q	+ 8q23.1-q24.11	22.87	MYC	[37]
9	-9P	- 9p21.1-p23	18.61	P14, p16, UBAPI, NGX6	[38–41]
11	+11q	+ 11q12.3-q13.5	16.49	Cyclin D1	[14]
	-11q	- 11q23.1-q25	29.79	TSLC1/IGSF4,	[42]
12	+12p	+ 12p12.1-p12.2	36.17		
		+12p11.21-p11.22	34.04		
	+12q	+ 12q13.11-q14.1	35.11	MDM2, STAT2	[43,44]
13	-13q	- 13q22.1-q31.3	18.61	EDNRB, LIG4	[45,46]
14	-14q	- 14q21.1-q21.3	25.00		
		- 14q24.3	27.13	AKTI/PKB, SIVA	[43]
15	+15q	+15q21.3-q25.3	26.67 E	DAPK2 (?)	[47]
16	-16q	- 16q13-q24.3	29.79	E-cadherin, BRD7	[48,49]
18	+18p	+18p	20.00 S		
	+18q	+18q21.1-q21.33	21.25 S		
20	+20q	+20q11.21-q13.13	30.00 E	PLUNC (?)	[50]

**Figure 3:**
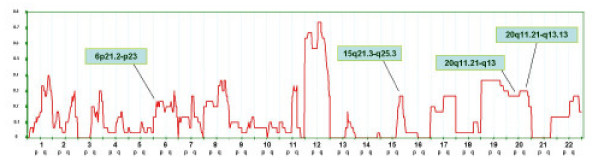
Frequency of chromosomal in 30 samples derived from patients with NPC known to contain EBV virus. The Y axis reports the frequency of chromosomal imbalances for individuals chromosome regions clustered into sections as described in the Materials and Methods. The X axis represents and ordered distribution of the chromosome analyzed.

Two studies reported the EBV status of a tumor for a total of 30 cases [10,17] In all cases, the tumors displayed evidence of EBV presence. Because this information supports with higher confidence the categorization of NPC to the WHO EBV-related tumors, we performed a separate meta-analysis only on these samples. The results were largely similar to those observed in the whole population analyzed (Figure 3). However, additional regions were identified with high frequency of imbalances that could be best explained by a higher sensitivity of the methodologies applied in these particular studies. Alternatively, it is possible that the other studies included cases of tumors not harboring EBV and, therefore, not canonically EBV-related. In this particular though unlikely case, these 4 hot spots could be considered DSA specifically linked to the EBV etiopathogenesis.

### Chromosomal imbalances according to WHO staging

**Figure 4:**
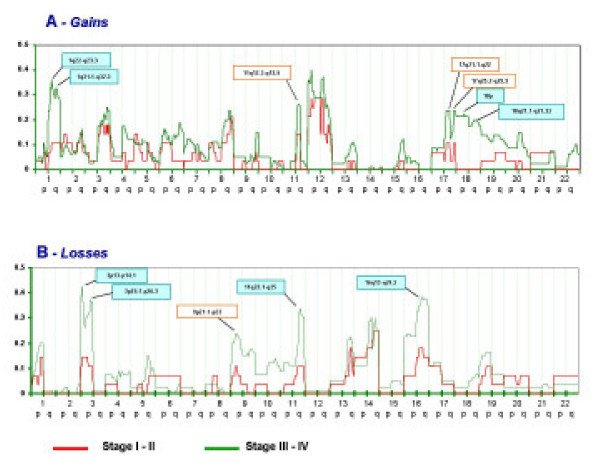
Frequency of chromosomal gains (panel A) or losses (panel B) in 108 samples derived from patients with NPC of which 28 were grouped as WHO stage I-II (red line) and 80 as WHO stage III-IV (green line). The Y axis reports the frequency of chromosomal imbalances for individuals chromosome regions clustered into sections as described in the Materials and Methods. The X axis represents and ordered distribution of the chromosome analyzed.

When the information was available, cases of NPC were grouped according to *WHO *staging [18]. One hundred-eight cases could be collected of which 28 were grouped as stage I-II and 80 as stage III-IV. Comparison of frequencies in chromosomal imbalances was carried between the two categories and their significance was examined based on a Fisher's exact test. In general, the same patterns of chromosomal gains or losses were seen in both categories with a trend to higher frequencies in advanced stages (Figure 4). Statistical analysis identified specific areas whose frequency of imbalances was significantly related to stage (Table 2). Of interest were also areas of gain such as 3p, 8q, 12p, 12q or loss such as 14p and 14q where a similar frequency was noted independently of stage suggesting that, possibly, this represent earlier and more fundamental chromosomal aberrations that occur early during NPC oncogenesis.

**Table 2:** Significantly different frequencies of chromosomal imbalances in early compared to advanced NPC.

	Selected Region	Stage (%)		Fisher's exact
			
		I-II (n = 28)	III-IV (n = 80)	
Gain	+1q22-q23.3	10.71	36.25	0.015
	+1q31.1-q32.3	10.71	32.50	0.015
	+18p	00.00	21.25	0.005
	+18q21.1-q21.33	00.00	20.00	0.010
Loss	-3p12.3-p14.1	14.29	42.50	0.010
	-3p25.1-p26.3	10.71	37.50	0.008
	-11q23.1-q25	10.71	33.75	0.026
	-16q13-q24.3	14.29	37.50	0.032

### Putative sub-grouping of NPC cases according to relatedness of chromosomal imbalances

**Figure 5:**
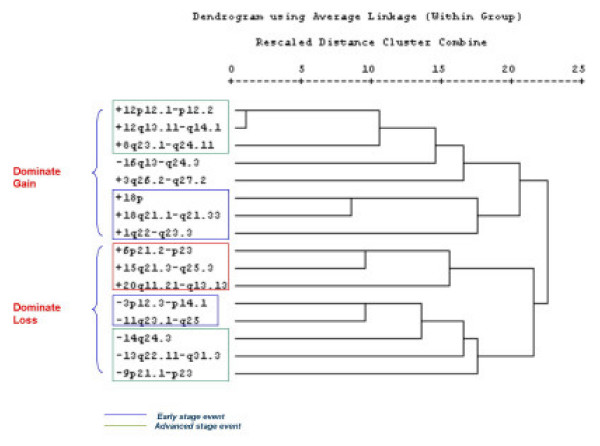
Correlation analysis based on clustering programs comparing the relatedness of the most frequently chromosomal imbalances identified in this study (frequency at least > 20%). Chromosomal regions representative of individual hot sports were selected as described in the material and methods. Distances are presented according to the φ 4 point correlation coefficient. Green boxes underline clusters including chromosomal aberrations found in early (Stage I-II) and late (Stage III-IV) NPC; blue boxes define clusters enriched with aberrations specific for late stage NPC and the red box defines chromosomal imbalances identified only by the studies in which the EBV status of the tumor was reported.

Clustering analysis was performed by combining gain and loss data according to a binomial mathematical measure in which lack of imbalances was assigned a value of 0 and gain or losses were indiscriminately assigned a value of one across the data set. Hot spots containing imbalances occurring more that 20% of the times in the population tested were then evaluated by selecting the single representative region within each hot spot portraying the highest frequency value. This data set was, then, combined and cluster analysis was performed (Figure 5). This explorative test identified two sub groups of NPC predominantly characterized by gains and the other by losses. In each category two subgroups could be observed one in which imbalances occurring in any stage of NPC were clustered together (green boxes) and those predominantly occurring in advanced stage NPC (blue boxes). Interestingly, a sub group of NPC was identified that included chromosomal imbalanced detected only in NPC cases for which there knowledge about EBV presence was suggesting that either these studies were characterized by a different level of resolution in identifying chromosomal imbalances or that the NPC cases included in these studies represented a biologically distinct subtype of cancer tightly linked to a EBV-related etiology while the cases included in the other studies represented a more heterogeneous population.

## Discussion

Meta-analyses are a powerful tool that allows the identification of patterns consistent in independent studies that, therefore, permits segregating true biological entities from artifactual findings related to biases associated with individual studies. The present meta-analysis of NPC cases explored the frequencies reported of chromosomal aberrations associated with NPC that could be detected with a low resolution assay such as CGH. Consistently present gains and losses could be identified which were defined as "hot spots". Although some of them appeared to occur with some reasonable frequency, none of them appeared to be particularly predominant with the most frequent chromosomal imbalances occurring at most in 30% of the cases evaluated. This phenomenon implies that NPC develops through the accumulation of multiple low-frequency genetic events. The presence of several genes known to be related to the etiopathogenesis of NPC in the hot spots identified further supports the conclusion that alterations in these genomic regions may play a prominent role in the oncogenesis. In addition, the identification of hot spots in which not know NPC-specific biomarker as been reported (Table 1) proposes other genomic regions of possible interest to be investigated for the identification of unknown cancer inducing factors.

Stage specific and incremental imbalances were observed with no hot spot identified in early cases (Stage I-II) that could not be seen in later cases (Stage III-IV) but several examples of incremental imbalances associated with advanced NPC. Surprisingly, unique imbalances were identified in cases in which the EBV status of the tumor was reported. Although it is conceivable that those cases in which EBV status was not reported may have represent disease taxonomy un-related to the EBV etiopathogenesis this possibility seems quite unlikely. First the cases analyzed were from an area of the World where EBV-associated NPC is endemic. Second, all cases in which the EBV status was evaluated, its presence turned out to be evident suggesting that also in the other cases the same results could be expected. It is, therefore, possible, that the two studies presenting this information [10,17]. were carried out adopting methodologies characterized by different level of sensitivity in detecting chromosomal imbalances. This question will need to be further addressed in future studies.

Interestingly, NPC appeared to segregate, based on cluster algorithms of relatedness, according to a general tendency toward chromosomal loss or gain. It is only, within category of loss or gain that imbalances present in early and late stage NPC segregated from those occurring at a later phase. This is reminiscent of the "tree model" of NPC pathogenesis that we previously reported [4]. Whether, this finding has a biological foundation or is related to a different sensitivity of CGH in reporting gain or losses will need also to be further explored.

Recently, Yan W et al [19] reported a novel study addressing chromosomal abnormalities associated with cervical nodal metastases in NPC. Since this information was not available at the time of our studies, we utilized this study to validate the prediction power of our results on an independent data set. In Yan's study, 23 samples of primary NPC were compared with 9 samples derived from lymph node metastases. A similar pattern of genomic imbalances was seen in primary and metastatic tumors reflecting their common clonal origin. Primary tumors were characterized by frequent gains of 5p, 12p, 12q and 18p and losses of 1p, 3p, 9q that identified hot-spots (frequency = 15%) as gains 12 12p and 12q and as losses in 1p, 3p, 9q, 14q and 16 q (Figure 2). In addition, the areas of frequent aberrations that did not appear as hot spots in the meta-analysis (< 15% frequency) were still characterized by higher frequency than in the direction (gain or loss) indicated by Yan's study. Thus, it appears that the results compiled by the meta-analysis could predict with high accuracy the findings observed in an independent set of individuals.

Chromosomal aberrations observed specifically in lymph nodal metastases in Yan's study included also losses in 9p, 16p, 17q, 20q, 21p, 21q and 22q and gains in 8q and 8p [19]. These findings diverged from the results of the meta-analysis based on primary NPC samples with only loss of 9p and gain of 8q that were represented by hot spots. In addition, loss of 16p was found in 100% on lymph nodal metastases, the meta-analysis results demonstrated that loss of 16p is more common in advanced stage primary NPC with a frequency below 15% in early stages and above 20% in stage III-IV. Thus, this region, may be a potential area of interest when looking at genes that might be associated with the metastatic potential of NPC and other cancers [20-24]. However, also in this case, when the results did not collimate the trends noted were in the same direction. Overall, this meta-analysis aimed at unifying other's results about chromosomal imbalances associated with NPC development and progression, identified consistent aberrations that could be identified in an independent study. This suggests that these chromosomal imbalances are strongly specific for the disease.

## Materials and methods

### Literature search

Searches were performed on PubMed and Medline using the following combination of search terms: "NPC", "Nasopharyngeal carcinoma", "and "CGH" and "comparative genome hybridization". In total, 12 studies were identified [8-13,17,25-29]., however, only 6 [8-12,17] were eligible for further analysis based on the following inclusion criteria: only NPC tumor biopsies were evaluated (studies using NPC cell lines and xenograft cell lines were excluded), the studies involved exclusively Southern Asian NPC patients, applied comparable Chromosome CGH analysis platforms, reported detailed CGH values using similar thresholds for definition of chromosomal loss or gain (if the normal DNA vs tumor DNA ratio was < 0.75 the region was considered as a chromosomal gain; if >1.20 it was considered a loss). A total of 188 NPC cases were identified from the 6 studies including 3 studies from Hong Kong, 2 from China, and 1 from Taiwan. Significant chromosomal imbalances were detected in 155 patients. World Health Organization (WHO) staging [18] was available in 108 cases; 28 belonged to early stage (stage I and II), 80 belonged to advanced stage (stage III and IV). EBV positive status was reported only in 30 patients from two studies.

### Data integration

CGH data derived from the 6 studies was standardized by dividing each chromosome arm into 20 sub-regions from the centromere toward each telomere. Chromosome gains and losses reported by the 6 studies were assigned to the relevant chromosomal region and gains or losses were recorded into two separate excel files reducing the information into a binary model (1 = chromosome imbalance, 0 = no imbalance). Chromosomal loss or gain were defined as previously stated according to a threshold signal intensity ratio between normal DNA over tumor DNA < 0.75 (gain) or > 1.20 (loss). WHO staging of NPC and EBV status were also entered in the data file.

### Definition of hot spot and Disease Status-Associated Imbalance (DSAI)

Regions with high frequency of chromosomal imbalances were considered 'hot spots". These were defined as chromosome regions with frequency imbalance observed in at least 15% of cases while the adjacent regions (boundaries of the hot spot) displayed a drop if frequency of at least 2%. The 15% value was arbitrarily selected based on the observation that the median frequency of chromosomal gains was 5.8% (1^st ^quartile = 2.7 and 3^rd ^quartile = 11.2) with an average of 7.7 ± 7.0 (Standard deviation, SD) and of losses was 3.7% (1^st ^quartile = 1.6 and 3^rd ^quartile = 8) with an average of 6.4 ± 7.4 (SD). A value of 15% included, therefore, chromosomal regions with a frequency of imbalances at least more than one SD above the mean and above at least the 3^rd ^quartile across the all data set.

Disease status associated imbalance (DSAI) corresponded to a chromosome region with a significantly high frequency of imbalances in clinically relevant subgroups of cases such as advanced versus early stage patients. The 1p, 16p, 19p, 19q, 22q regions and the whole Y chromosome were not analyzed because they have been described to yield false positives results in CGH analysis [8,11]. Finally, the X chromosome was also not analyzed due to a rare prevalence of events associated with it and lack of information about the patient's gender.

### Statistical analysis

Chromosomal gains and losses were separately profiled for all chromosomal regions previously defined arms by assigning a binary value of 1 or 0 according to presence or absence of imbalance for that region in each sample studied. Frequency of imbalances for each chromosomal region analyzed was then calculated and graphically visualized. Hot spots were selected for further analysis. Additionally, the frequency of chromosomal imbalances observed in studies reporting the EBV-status of tumors was also separately analyzed because of the higher confidence that these tumors represented classical EBV-induced NPC. A two-tailed Fisher's exact test was applied to explore the strength of relationships between given chromosomal imbalances and stages of NPC in order to identify DSAI. Based on hot spots and DSAI, a hierarchical clustering analysis [30] was performed to explore possible correlations among distinct chromosomal regions where imbalances were frequent. A flow chart representing the procedure of the meta-analysis is shown in Figure 1.

### Clustering algorithms

Hot spots with a frequency above 205 were selected for clustering analysis to identify potential subgroups of NPC with a defined pattern of genetic imbalances. Within in each spot a representative region was selected by choosing the region associated with the highest frequency on imbalances. Data from each of the selected region was gathered in a separate data base assigning a value of 0 to individual cases where no genetic imbalances were observed and of 1 to either losses or gains. In this fashion chromosomal imbalances could be clustered independently of their genetic tendency toward loss or gain. Hierarchical clustering was then carried on SPSS 10.0 based on hot-spot ranking values giving an approximation of relationship among various imbalances measured according to the Φ 4 point correlation coefficient.

This article was partly supported by a grant (30371535/C030310) from National Natural Science Foundation of China.
